# A study of impulsivity as a predictor of problematic internet use in university students with disabilities

**DOI:** 10.3389/fpsyt.2024.1443289

**Published:** 2024-10-22

**Authors:** María J. Pino, Carlos Herruzo, Valentina Lucena, Yolanda Trenados, Javier Herruzo

**Affiliations:** ^1^ Department of Psychology, University of Cordoba, Cordoba, Spain; ^2^ Departament of Social, Psychology, Social Work and Social Services, and Social Anthropology, University of Malaga, Malaga, Spain

**Keywords:** problematic internet use, impulsivity, disability, university student, risk factors

## Abstract

**Introduction:**

The role played by impulsivity in problematic internet use (PIU) is the object of much debate among researchers. Some studies emphasize its importance, while others suggest mental distress or personality traits may be more crucial. More research into the issue is clearly needed—especially in at-risk populations like people with disabilities. The objectives of this study were therefore to investigate the relationship between PIU and impulsivity among university students with disabilities, and to develop a specific predictive model for this group that would include psychological and life-functioning variables.

**Methods:**

A cross-sectional design was used with a sample of 240 Spanish university students with disabilities from seven universities. Several instruments were used for data collection: a sociodemographic questionnaire, the Internet Addiction Test (IAT), the Clinical Outcomes in Routine Evaluation-Outcome Measure (CORE-OM), and NEO-FFI-R.

**Results:**

The results indicated that impulsivity is significantly correlated with PIU, as indeed it also is with other factors like conscientiousness and social functioning problems. The study's regression model explained nearly 50% of PIU variance, with impulsivity, personality traits, wellness and health indicators, and social media usage as predictive variables.

**Discussion:**

This suggests that interventions should consider these psychological and lifestyle variables as a means of mitigating PIU risks in students with disabilities. The findings identify a need for further longitudinal studies to understand the causality and develop targeted prevention strategies.

## Introduction

Over the last twenty years, interest in problematic internet use (PIU) has grown exponentially, in parallel with the global increase in the use of internet. According to the Digital Report of 2024 (1), internet is now accessed by 62.2% of the population, with percentages rising to almost 100% in some regions. PIU refers to an inability to control the use of the internet resulting in psychological, social, school or work difficulties in a person’s life (2, 3). Systematic reviews have already been conducted to explore PIU in relation to different psychological variables, patterns of use and lifestyles (4), and in different populations. Some of these studies have been cross-cultural (5). However, research gaps still persist with regard to potentially more vulnerable groups, such as people with disabilities (6).

When addressing the use of the internet by people with disabilities, two key factors have been identified in the scientific literature: on the one hand, this group’s greater vulnerability to the negative consequences of PIU (6), and on the other, internet’s ability to mitigate the barriers that hinder the inclusion of people with functional diversity. Online communication seems to be an effective tool for fostering adequate social support and creating conditions that promote psychological health, thus counteracting social interaction difficulties resulting from disability (7–9).

As has been pointed out on several occasions (6, 7, 10), however, the literature on PIU and its impact on people with disabilities remains scarce both in Europe and elsewhere. To date, studies have mainly focused on intellectual disability or cyberbullying, leaving a significant gap in our understanding of the diversity and complexity of the whole disability domain (11). Moreover, although a lower prevalence of PIU has been observed in people with disabilities compared to the general reference population, the negative consequences such people experience in terms of their well-being and psychological health are more severe (6). It has not yet been confirmed, however, whether the PIU risk and vulnerability variables identified in the general population are extrapolatable to people with disabilities.

With regard to PIU risk variables in the population without disabilities, a recent systematic review by Sánchez-Fernández et al. (4) identified 10 predictor variables for PIU. These were classified into three groups: patterns of use, psychological variables, and lifestyles. Among the variables related to patterns of use, time spent online and engagement in online gaming were identified as potential PIU risk factors. Of the psychological variables, depression, negative affect, life stress, maladaptive cognitions, and impulsivity were found to be risk factors, while conscientiousness was a protective factor. Finally, poor sleep quality and substance use (alcohol and drugs) were identified as lifestyle variables that constitute risk factors.

One particular risk variable identified in the literature—impulsivity—has been cited in several of the most important theoretical models that have emerged from research into PIU. These include the I-PACE model (12, 13), Young’s internet addiction model (14), and models proposed by Demetrovics et al. (15) and LaRose (16)—self-regulation models which interpret PIU as a self-regulation deficiency problem. This approach is also found in one of the most recent research developments, known as the behavioral economics model (17–19).

Impulsivity has been defined as a trait characterized by the carrying out of unplanned actions which, although rewarding, are often inappropriate or inordinately risky for the situation at hand and may result in undesired consequences (20, 21). It is recognized as a multidimensional attribute that encompasses five key dimensions: negative and positive urgency (i.e., reacting hastily to intense emotions), lack of premeditation (i.e., acting without contemplating possible consequences); lack of perseverance (difficulty in maintaining concentration in the face of tasks perceived as tedious or complicated), and sensation seeking (the desire to engage in stimulating activities) (22).

On the other hand, researchers like Fineberg et al. (23) and Verdejo-García et al. (24) have identified three essential neurocognitive components of impulsivity: 1) the inability to inhibit dominant motor or cognitive responses, 2) the preference for smaller, immediate rewards over more significant, long-term rewards (reward discounting), and 3) reflective impulsivity, which manifests itself in the difficulty to adequately evaluate options or take reasonable risks and the tendency to ignore relevant information when making decisions, often leading to disadvantageous choices.

The relationship between impulsivity and addictive behaviors has been a subject of study in the fields of both “non-substance” and “substance use” addictions. Although a considerable amount of evidence has accumulated in this regard, results are not consistent and further research is still required to fully understand these dynamics (4, 25).

In the case of substance-related addictions, for example, impulsivity has been linked to early onset of use, transition to abuse and dependence, and to maintenance and relapse (24, 26–28). In behavioral addictions, such as online gambling, problematic internet use, gambling, exercise addiction, and compulsive shopping, higher levels of impulsivity are associated with higher rates of disorders (28–39).

Several studies into PIU have explored the factors influencing this problem, generating findings that highlight the role of impulsivity, although other works minimize its importance. Zhang (40), for example, found that impulsivity mediates the relationship between PIU and neuroticism, suggesting its relevance. Wang et al. (41) found that effortful control and impulsivity were related to PIU severity. Similar conclusions were drawn by Salehi, et al. (42) and Bernal-Ruiz and Rosa-Alcázar (43). However, other findings suggest that impulsivity may not be the main factor in the development of PIU (44, 45). Studies like that of Yücens and Üzer (46) suggest that mental distress may be more important than impulsivity. Others, like that of Zadra et al. (47), conducted using a large community sample, suggest that personality characteristics better explain PIU than impulsivity per se.

In short, more research is needed on the relationship between impulsivity and PIU, especially in populations that may be at risk. People with disabilities, for example, constitute a particularly vulnerable group that has received very little attention in the literature. In normative populations, it has been seen that university students may also be a group particularly vulnerable to PIU due to their unsupervised access to the internet and responsibility for their own time management. The prevalence of PIU among college students can be high and is associated with a variety of negative consequences, from psychiatric disorders to addictive behaviors such as pathological gambling (20, 23, 48–52).

It is therefore crucial to investigate whether the abovementioned risks are replicated in university students with disabilities, with special attention to the role of impulsivity—a variable which, according to the literature, appears to be closely linked to PIU. Accordingly, the objective of this paper was to investigate the relationship between PIU and impulsivity in university students with disabilities, and to develop a specific predictive model for this group that includes psychological and life-functioning variables.

## Materials and methods

### Sampling and participants

A sample group comprising a total of 240 Spanish university students with disabilities was selected from the users of the students with disabilities support services of seven universities (UNED–Spain's national distance learning university, University of Valencia, University of Cadiz, University of Malaga, University of Jaen, University of La Laguna and University of Barcelona). In order to make use of these services, students had to have a percentage of disability of more than 33%, accredited by the government's social and health services. 37% of the sample had a motor disability, 19% a sensory disability (auditory or visual), and 44% other disabilities such as chronic illness (excluding intellectual disabilities). 23% were born with a disability and the remaining 77% had an acquired disability, while 55% of the sample were women and 45% were men. The average age was 43.37 years (SD = 12.73) (the average age according to a study conducted throughout Spain by the State Disability Observatory (53) is 38.7; being in males slightly higher 39.7). The sample was recruited by means of an invitation to participate delivered through the services for students with disabilities at the above-cited universities. 90% of the participants were doing bachelor’s degrees and 10% master's degrees. 26% were students of Humanities; 40% of Social, Economic and Legal Sciences; and 33% of Health, Sciences and Technologies (ICT).

### Instruments

An *ad hoc* questionnaire was developed, containing several instruments.

Sociodemographic and Internet Use Questionnaire: This questionnaire collected information on gender, age, educational background, disability status, and internet usage habits, including the number of hours spent online and the percentage of time allocated to different activities such as leisure, work, and studies.

Internet Addiction Test (IAT): Adapted for Spanish speakers by Carbonell et al. (54) and validated in Spain by Fernández-Villa, et al. (55), this test evaluates the extent to which internet use impacts different aspects of daily life, such as social interactions, productivity, and emotional well-being. The test consists of 20 items rated on a Likert scale from 0 to 5, with higher scores indicating greater addiction severity. A cutoff score of 40 was used to classify participants as problematic users. The short version of the test, called IAT-12 and developed by Pino et al. (56), was validated for people with disabilities by Pino et al. (10) The internal consistency coefficient (Cronbach's alpha) was close to 0.90.

The Likes Questionnaire: Additional items were included after the IAT to assess the participants' behaviors related to the seeking of validation on social networks. This included things like feelings triggered by not receiving enough "likes" and the frequency with which people checked their follower counts. These items showed a significant correlation with the Addiction to Social Networks Questionnaire (r=.493, p<.001), with an internal consistency (alpha) coefficient of 0.752. The Addiction to Social Networks Questionnaire was validated in Spain by Casas et al. (57).

Clinical Outcomes in Routine Evaluation-Outcome Measure (CORE-OM), by Evans et al. (58), adapted for Spanish populations by Trujillo et al. (59). This is a self-report questionnaire consisting of 34 items that assess the subject's condition based on four dimensions: subjective well-being/discomfort (4 items); problems/symptoms (12 items, measuring anxiety, depression, trauma, and physical symptoms); life functioning (12 items, assessing intimate relationships, social relationships, and levels of daily functioning); and risk (4 items serving as clinical indicators of suicide attempts and self-harm, and 2 items to predict acts of aggression against others). Mean scores below 1 indicate healthy levels, except on the risk scale (<.35). The psychometric properties of this test demonstrated acceptable levels of internal consistency (Cronbach alpha values between 0.75 and 0.90) and sensitivity in the measurements obtained (58). The questionnaire has been used in numerous clinical settings (60, 61) and with university populations (60, 62). In addition, this test has shown convergent validity with the Beck Depression Inventory II (BDI-II) (63) and the Symptom Checklist-90-Revised (SCL90-R) (64).NEO-PIR: NEO Personality Inventory-Revised (NEO PI-R) (65), and its abridged version (NEO-FFI-R). The last test comprises a total of 60 items and has a 5-choice Likert-type response format (1.- strongly disagree and 5.- strongly agree). The questionnaire is based on the Big Five model and considers five main factors: 1) neuroticism (N): identifying individuals prone to psychological distress, unrealistic ideas, excessive cravings or urges and non-adaptive coping responses; 2) extraversion (E): evaluating the amount and intensity of interaction between people, their level of activity, their need for stimuli and their capacity for enjoyment; 3) openness to experience (O): assessing the active seeking and valuing of experience itself, with individuals presenting tolerance and exploration of the unknown; 4) agreeableness (A): assessing the quality of the individual's interpersonal orientation along a continuum from compassion to rivalry of thoughts, feelings, and actions; and 5) conscientiousness (C): assessing the individual's degree of organization, perseverance, and motivation in goal-directed behavior. Impulsivity and sensation seeking were evaluated using the 16 NEO-PIR items (8 for each dimension) that specifically address these dimensions. For example, for impulsivity "I have little difficulty resisting temptation” or "I rarely give in to momentary impulses", and for sensation seeking "I often seek exciting sensations" or "I love the thrill of roller coasters at amusement parks". The internal consistency alpha index values were acceptable (0.86 to 0.92). In the Spanish version (66), all NEO PIR's core scales achieved excellent reliability coefficients (r ≥ 0.85). The value for the specific scale of “impulsivity” was 0.57 and “sensation seeking” 0.56 (66). Regarding the NEO-FFI-R, this instrument has been validated in Spain by several authors (67–70). The alpha reliability of the NEO-FFI-R in this version were, respectively: Neuroticism (0.90), Extraversion (0.88), Openness (0.88), Responsibility (0.89) and Agreeableness (0.86).

### Data collection

The study adhered to the principles outlined in the Declaration of Helsinki and received approval from the Institutional Review Board of the Andalusian Regional Government (Ethics Committee). Following approval, the students with disabilities support services of the participating universities were contacted. These services distributed an email to the individuals listed in their databases containing a link to a website inviting them to participate in research on internet use. In the email and on the first page of the questionnaire it was explicitly stated that participation implied consent for the researchers to use the participants’ responses exclusively for research purposes, thus ensuring confidentiality, and that no additional data other than the survey responses would be collected.

### Statistical analysis

Pearson correlation coefficients were employed to examine the relationships between impulsivity, internet use, and other variables. Two-way ANOVA tests were used to investigate differences related to type or origin of disability. Prediction of problematic internet use (PIU) was addressed through multiple (forward stepwise) linear regression analysis, with predictors including impulsivity, sensation seeking, the Big Five personality factors (neuroticism, extraversion, openness, agreeableness, conscientiousness), various indicators of wellness and health (anxiety, depression, physical symptoms, traumatic symptoms, problems of daily functioning, problems of social relationship functioning, problems of social support functioning), social media usage (likes), gender and age.

## Results

Initially, Pearson correlation coefficients were calculated between PIU (IAT) and impulsivity, with a correlation of 0.378 (*p* <.001) and each predictor variable. Appendix I shows the correlations matrix, with the values for these variables, encompassing sensation seeking, the Big Five personality factors (neuroticism, extraversion, openness, agreeableness, conscientiousness), various indicators of wellness and health (anxiety, depression, physical symptoms, traumatic symptoms, daily functioning problems, social relationship functioning problems, social support functioning problems), social media usage (likes), gender and age. With the exception of sensation seeking and openness, all factors displayed significant correlations with PIU, ranging from 0.171 (extraversion) to 0.504. Impulsivity also showed correlation with all factors except openness [ranging from 0.142 to -0.498 (conscientiousness)]. Hereafter, two separate ANOVAs were conducted to explore the relationship between "impulsivity" and "disability", one categorized by type of disability (F[2.223]=1.07; *p=*.344) and the other by origin (t.[1,209]=.527; *p=*.468). No statistically significant differences were detected in either case.

The impact of impulsivity on PIU was further examined through multiple linear regression analysis, using scores from the Internet Addiction Test (IAT) as the dependent variable (see **Table 1**). Here, impulsivity, personality traits, wellness and health indicators, and social media usage were included as predictive variables. A model comprising 7 variables was derived, with impulsivity identified as a risk factor. The beta value for impulsivity was 0.122, indicating a small effect size according to Cohen (71). The R2 value of the model was 0.495 (Adjusted R square=.477), with an estimation standard error of 8.92834, and a Durbin-Watson index of 2.122 (close to 2.000). As established by Cohen (71), R2 values equal to or greater than 0.26 suggest a large effect size. The ANOVA for the model yielded F=28.51; *p*<.001.

**Table 1:** Linear multiple (Forward) regression model for Internet Addiction Test (IAT).

Model	Non-Standardized Coefficients		Standardized Coefficients	t	*p*	
	B	Standard Error	Beta			VIF
(Constant)	8.644	2.298		3.762	<.001	
Likes	3.742	.497	.392	7.533	<.001	1,091
Conscientiousness	-5.510	1.103	-.315	-4.996	<.001	1,605
Relationship problems	3.359	1.065	.206	3.154	.002	1,721
Daily functioning problems	-3.994	1.148	-.229	-3.480	<.001	1,751
Openness	2.281	.885	.130	2.577	.011	1,024
Social support problems	2.433	1.025	.162	2.374	.019	1,871
Impulsivity	2.146	1.030	.122	2.084	.038	1,375

Dependent Variable: IAT

VIF, variance inflation factor

## Discussion

The objectives of this study were to investigate the relationship between impulsivity and PIU in university students with disabilities, and to develop a predictive model of PIU, specific to this group, that would include psychological and life functioning variables.

Regarding the first objective, our results indicate that in the university population with disabilities there is a positive relationship of intermediate magnitude between impulsivity and PIU (71) which coincides with that found in students without disabilities. Previous studies, such as the meta-analysis by Koo and Kwon (72), support this association, as do other studies conducted with both normative university populations (42, 43, 45, 73, 74) and non-university students (32, 38, 41). It is therefore important to consider impulsivity as a risk variable for problematic internet use in the university population, and the present study provides new evidence regarding a vulnerable group that has to date been under-researched.

Regarding the second objective, the predictive model for PIU, elaborated using multiple linear regression analysis, was able to explain almost 50% of the variance based on impulsivity, social network use, personality, and life functioning variables. The negative correlation found between PIU and the personality trait “conscientiousness” is in line with other studies in which conscientiousness has been found to be a protective factor against PIU (4, 75–77). In our model, variables related to life functioning also stood out: according to the results obtained, social relationship functioning problems and social support functioning problems both act as risk factors for PIU. These results appear to concur with the findings of Weiser (78), who observed that individuals who use the internet to satisfy their social needs or as a means of personal communication are more at risk of developing internet addiction (78).

In our regression model, however, there are two variables—openness and daily functioning—that require a more detailed explanation.

Openness appears as a risk variable in the model, despite not correlating significantly with PIU (see Appendix I). Its inclusion in the model following the multiple regression analysis could indicate that, even though the trait itself does not predict PIU, it does carry weight when accompanied by the other factors in the model, such as impulsivity. That is to say, openness to experience together with impulsivity and low conscientiousness may be better predictors of PIU than openness alone. These results reinforce the idea, put forward by several authors, that it is necessary to study personality traits in relation to PIU as patterns rather than considering each trait in isolation (79, 80).

The regression model also showed daily functioning problems to be a protective factor, even though, again, no positive correlation was found with PIU. While apparently contradictory, this could be understood in the context of the vital functioning of the population with disabilities with whom the study was conducted. At the individual level, this factor presents a positive correlation of moderate magnitude (*r=*.306; *p*<.001) with PIU, meaning that problematic internet users tend to have greater problems of daily functioning, just as they have greater problems of social relationships or social support. This concurs with other studies in the literature which report that people with PIU generally present more psychological problems (4). However, when daily functioning skills are considered not in isolation but in interaction with high impulsivity, openness to experience and low conscientiousness, they seem to contribute to a greater tendency to engage in problematic internet use. This could mean that for someone who is impulsive and has low responsibility, that person's ability to achieve things (daily functioning) may be used to reduce deficits in social interaction and support through internet activities, which are more accessible. In other words, when such a person has problems with social support and relationships, they may seek to escape from that discomfort by engaging in internet activities, and their ability to pursue a goal could thus be used as a mechanism for doing so. Taken together with greater impulsivity and less responsibility, this would lead the person to become increasingly involved in those escape activities, coinciding with their setting of new goals such as getting followers or "likes"—indicated in turn by the "like" factor (another of the risk factors that appear in the model). These results concur with the findings of Pino et al. (10) in that they suggest that college students with disabilities who reported using the internet primarily for social networking and other recreational purposes have a much higher proportion of problematic users than those whose primary use of the internet is for studies and work. In this regard, the results obtained by Herruzo et al. (6) show that people with disabilities who have PIU suffer significantly more subjective distress than those whose use of such technology is more controlled. In the long run, this may lead to greater seclusion, as demonstrated by Duplaga and Sluzc (81), who found that PIU is conducive to isolation.

On the other hand, the results obtained in this study would fit in with what is predicted in the behavioral economics model (17–19), in which the so-called "reinforcer pathology" occurs in the context of diminished reinforcement of alternative activities, resulting in a higher valuation of addictive behavior relative to available alternative activities. The diminished availability of alternative rewards in their environment could thus lead to a higher level of PIU among people with disabilities (82). That is to say, obtaining reinforcement from, for example, social support or intimate relationships in a non-virtual environment entails more effort, time, and resources for persons with disabilities, so they are generally deprived of such reinforcement. As a consequence, PIU may be easier to find in this group, because the internet would provide immediate reinforcement in these areas of life and would facilitate their access to such reinforcement. Under such circumstances, the valuation of the Internet by students with disabilities would increase due to a change in the cost/benefit ratios of both the internet and its alternatives. PIU is made more problematic for people with disabilities precisely because of the reinforcement-deprived environment associated with the inaccessibility of alternative activities (82). In recent years, Spanish universities have provided counselling and psycho-educational services and programs for students with disabilities, which has led to the inclusion of this group in the academic activities offered by the university. However, very few students with disabilities admit to taking part in social activities or meetings. And it is these kinds of non-instrumental activities that are particularly important for their socialization. University students with disabilities feel that they have more difficulties than their peers in socializing with their peers and feel more comfortable in distance learning, online (83).

Regarding the limitations of the present study, it must be acknowledged that, since it is a cross-sectional study, it is impossible to know the directionality of the relationships or whether a feedback mechanism is produced. Longitudinal studies and more complex analyses would therefore have to be carried out to explore whether impulsivity plays a mediating role in the relationship between PIU and the factors present in our regression model. Another limitation of the study is the size of the sample. It would be interesting to increase the sample size sufficiently to be able to study the differences between the different types of disability in more depth and the protective effect of age. In principle, age seems to act as a protective factor, but this could be a cohort effect due to the lower exposure of older generations or the reduction of impulsivity acquired over time.

With a view to future lines of research, the results obtained in this study could provide a basis from which to explore the generalization of existing findings on impulsivity and addictions to university students with non-intellectual disabilities. Impulsivity has been associated with the onset of any type of addictive behavior, involving as it does difficulties in planning and predicting, reduced perseverance and a high level of desire for immediate gratification (30, 73), so all of these variables should be explored in people with disabilities.

In conclusion, this study has confirmed with university students with disability something that has been found in previous studies with other populations: the need to pay attention to impulsivity as a risk factor for the development of PIU. It also shows that impulsivity, when accompanied by other risk factors such as social isolation, openness, problems in social relationships and low conscientiousness, can predict PIU, explaining 49.5% of variability.

This paper provides a strong argument for taking these risk and protective factors into account when addressing prevention policies for university students with disabilities—a continually growing group of people who often face more environmental challenges than their without disabilities peers (84), and who, although PIU is less prevalent among them than among other university students, are nevertheless more vulnerable to psychological and well-being problems than the latter (56, 85, 86).

## Data Availability

The original contributions presented in the study are included in the article/supplementary material. Further inquiries can be directed to the corresponding author.

## Appendix I

**Table:** Correlation matrix between the study variables.

	1	2	3	4	5	6	7	8	9	10	11	12	13	14	15	16	17	18	19	20
**1**	–																			
**2**	.378**	–																		
**3**	.078	.142*	–																	
**4**	.377**	.464**	-.047	–																
**5**	-.171*	-.198**	.416**	-.499**	–															
**6**	.041	-.031	.060	-.137*	.206**	–														
**7**	-.177**	-.256**	-.164*	-.248**	.199**	.127	–													
**8**	-.471**	-.498**	.080	-.463**	.439**	.097	.324**	–												
**9**	.504**	.145*	.102	.265**	-.038	-.085	-.034	-.189**	–											
**10**	.391**	.373**	-.045	.635**	-.361**	-.027	-.125	-.297**	.273**	–										
**11**	.282**	.374**	-.070	.601**	-,353**	-.044	-.222**	-.355**	.174**	.762**	–									
**12**	.098	.168*	-.082	.252**	-.216**	.051	.001	-.091	.108	.498**	.500**	–								
**13**	.335**	.360**	.061	.475**	-.183**	.123	-.228**	-.243**	.193**	.642**	.654**	.358**	–							
**14**	.221**	.306**	-.068	.586**	-.533**	-.071	-.178**	-.490**	.159*	.532**	.577**	.296**	.369**	–						
**15**	.342**	.201**	-.076	.604**	-.367**	-.100	-.253**	-.321**	.196**	.547**	.575**	.205**	.493**	.557**	–					
**16**	.419**	.307**	-.064	.575**	-.400**	-.121	-.266**	-.367**	.243**	.677**	.705**	.348**	.592**	.452**	.589**	–				
**17**	-.243**	-.121	-.190**	-.175**	.070	.112	.072	.163*	-.227**	-.186**	-.092	.029	-.175**	-.053	-.073	-.152*	–			
**18**	.060	.004	-.126	-.187**	-.095	.031	.014	-.038	.065	.104	.111	.182**	.035	.052	-.078	.038	-.186**	–		
**19**	.113	.091	-.026	.171*	-.204**	-.064	-.081	-.131*	.037	.150*	.091	.016	.102	.229**	.151*	.168*	.087	.005	–	
**20**	-.063	.050	-.087	.037	-.106	-.079	-.018	-.039	-.071	.071	.069	.209**	-.099	.138*	.097	.022	.494**	.021	.260**	–

**. The correlation is significant at the 0.01 level (bilateral).

*. The correlation is significant at the 0.05 level (bilateral).

1= IAT (Internet Addiction Test); 2= Impulsivity (NEO-PI-R); 3= Sensation seeking (NEO-PI-R); 4= Neuroticism (NEO-FFI-R); 5= Extraversion (NEO-FFI-R); 6= Openness (NEO-FFI-R); 7= Agreeableness (NEO-FFI-R); 8= Conscientiousness (NEO-FFI-R); 9= Likes; 10= Anxiety (CORE-OM); 11= Depression (CORE-OM); 12= Physical symptoms (CORE-OM); 13= Trauma symptoms (CORE-OM); 14= Life functioning (CORE-OM); 15= Friendship support functioning (CORE-OM); 16= Social relationship functioning (CORE-OM); 17= Age; 18= Gender; 19= Type of disability; 20= Origin.
